# 

**Published:** 1971-01

**Authors:** 


### BRISTOL MEDICO-CHIRURGICAL JOURNAL

RECEIVED
MAR 31 1971
National Library of Medicine

JANUARY 1971

VOL. 86 (i) No. 317

